# An audit of licenced Zambian diagnostic imaging equipment and personnel

**DOI:** 10.11604/pamj.2020.36.32.21043

**Published:** 2020-05-22

**Authors:** Chitani Mbewe, Pascalina Chanda-Kapata, Veronica Sunkutu-Sichizya, Nason Lambwe, Nataliya Yakovlyeva, Masauso Chirwa, Birhanu Ayele, Richard Denys Pitcher

**Affiliations:** 1 Division of Radiodiagnosis, Department of Medical Imaging and Clinical Oncology, Faculty of Medicine and Health Sciences, Stellenbosch University and Tygerberg Hospital, Cape Town, South Africa; 2 Public Health and Research, Ministry of Health (MOH), Zambia, Haille Selassie Avenue, Ndeke House, P.O. Box 30205, Lusaka, Zambia; 3 Radiology Department, University Teaching Hospital, Nationalist Road, Private Bag RW1X, Ridgeway, Lusaka, Zambia; 4 Nuclear Medicine Unit of the Radiology Department, University Teaching Hospital, Nationalist Road, Private Bag RW1X, Ridgeway, Lusaka, Zambia; 5 Department of Radiology, Copperbelt University (CBU) and Ndola Teaching Hospital, Broadway Avenue, Ndola, Zambia; 6 Department of Social Sciences and Research, University of Zambia, Lusaka, Zambia / The University of Warwick, Coventry, CV4 7AL, United Kingdom; 7 Division of Epidemiology and Biostatistics, Faculty of Medicine and Health Sciences, Stellenbosch University, Cape Town, South Africa

**Keywords:** Zambia, low-middle income country, diagnostic radiology resources

## Abstract

**Introduction:**

Estimates indicate that two-thirds of the world's population lack adequate access to basic medical imaging services integral to universal health coverage (UHC). Furthermore, sparse country-level radiological resource statistics exist and there is scant appreciation of how such data reflect healthcare access. The World Health Organisation posits that one X-ray and ultrasound unit for every 50,000 people will meet 90% of global imaging demands. This study aimed to conduct a comprehensive review of licensed Zambian radiological equipment and human resources.

**Methods:**

An audit of licensed imaging resources, using the national updated Radiation Protection Authority and Health Professions Council of Zambia databases. Resources were quantified as units or personnel per million people, stratified by imaging modality, profession, province and healthcare sector, then compared with published Southern African data.

**Results:**

Over half of all equipment (153/283 units, 54%) and almost two thirds of all radiation workers (556/913, 61%) are in two of ten provinces, serving one third of the population (5.49/16.4, 33.5%). Three-quarters of the national equipment inventory (212/283 units, 75%) and nearly ninety percent of registered radiation workers (800/913, 88%) are in the public sector, serving 96% of the population. Southern African country-level public-sector imaging resources principally reflect national per capita healthcare spending.

**Conclusion:**

To achieve equitable imaging access pivotal for UHC, Zambia will need a more homogeneous distribution of specialised radiological resources tailored to remedy disparities between healthcare sectors and provincial regions. Analyses of licenced radiology resources at country level can serve as a benchmark for medium-term radiological planning.

## Introduction

While healthcare workers worldwide embrace the notion of universal health coverage (UHC) as reflected in the United Nations (UN) 2030 Agenda for Sustainable Development, there is growing scrutiny of global radiological resources [1-3]. Health is centrally positioned within the UN 2030 Sustainable Development Goals (SDGs) and is addressed in one comprehensive goal (SDG 3), comprising thirteen targets [2, 4]. Diagnostic imaging has the potential to contribute to achieving six of the SDG health targets. It can assist in reducing maternal and child mortality, deaths due to road traffic accidents and non-communicable disease (NCD) mortality, as well curtailing the TB/HIV pandemics, and improving reproductive health services [5]. Additionally, global radiological services would be substantially enhanced by the realization of a further two SDG health targets, namely improving coverage of essential health services and promoting training of health workers [6]. Thus, there exists considerable inter-dependence between health-related SDG targets and the extension of global imaging services [4, 5].

**Figure 1:**
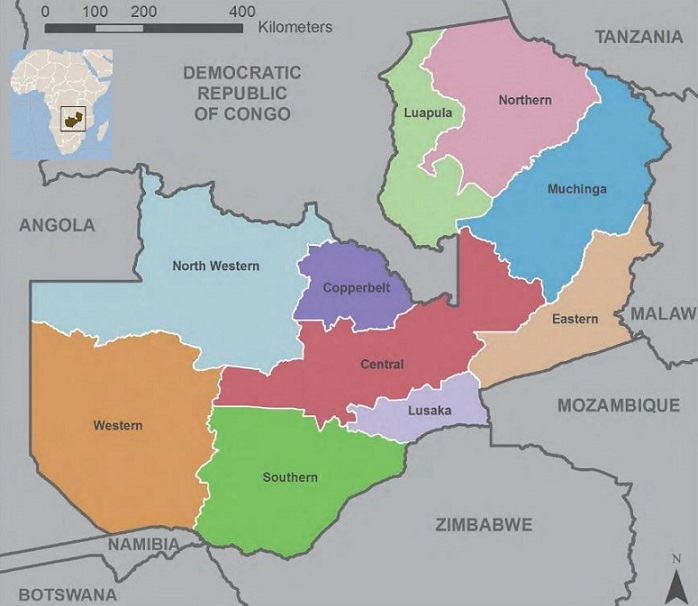
Map of Zambia's administrative provinces

Radiology is increasingly acknowledged as a pivotal diagnostic tool [4, 7]. The World Health Organization (WHO) recognizes basic diagnostic imaging services as vital to any healthcare system and suggests that one basic X-ray and ultrasound unit for every 50,000 people (or 20 units per million) would address 90% of global imaging needs [8-10]. However, worldwide shortages of imaging equipment and personnel, as well as inequalities in access to services, are increasingly cited as barriers to UHC [11, 12]. More than half the world's population lack access to even basic radiology services [13]. Furthermore, disparities in basic imaging services between and within nations are perceived as exacerbating health care inequalities [4, 8, 9]. The compilation and dissemination of country level statistics on medical devices was mandated by the World Health Assembly resolution WHA 60.29 of 2007 [14]. However, there are scant published data on licenced diagnostic imaging equipment resources at national level [8, 9, 15]. WHO estimates of medical imaging resources at country-level are based on questionnaire surveys of member countries [10, 16]. However, these data are confined to high-end imaging modalities and do not include basic equipment such as general radiography and fluoroscopy [8, 9]. Worldwide, governments are experiencing increasing pressure to fund essential public-sector services, including healthcare [4, 8, 17]. The achievement of UHC poses unique challenges for each country, and is a function of the national economy, health budget, existing healthcare infrastructure, demographics, burden of disease, and donor funding [9, 17, 18]. This is certainly true for Zambia, a 752614 square kilometre, land-locked, lower-middle income Sub-Saharan African (SSA) country. Zambia, faces substantial healthcare challenges, including dual HIV and PTB pandemics, a high infant mortality rate, and increasing trauma and NCDs [19-22]. Zambia's predominantly rural population (56.5%) of approximately 16.4 million people (2017) has a median age of 17 years and a density of 22 people/km2 [22, 23]. Zambia has ten administrative provinces, being Central, Copperbelt, Eastern, Luapula, Lusaka, Muchinga, Northern, North-western, Southern and Western provinces (Figure 1) [19, 23].

Zambia, Africa's second-largest copper producer, achieved middle-income country status in 2011 during a decade (2004-2014) in which the country had one of the world's fastest growing economies, averaging 7.4% per year [24, 25]. Since 2015, growth has slowed due to falling copper prices, reduced power generation, and currency volatility [26]. Approximately 5% of GDP is spent on health, equating to $ 195 USD per capita in 2014 and $ 56.5 USD in 2016 [27, 28]. There are four main healthcare providers, namely the government, faith-based (not-for-profit) organizations, the mine hospitals, and private enterprise (for-profit). The government and faith-run institutions are considered public sector entities, servicing more than 90% of the population [22, 29-31]. The country's public health service mirrors the political administration, with 10 provinces and 105 districts. There are three tiers of public healthcare. The first provides preventive and primary services at Health Posts (n = 953), Rural Health Centres (n = 1839) and District Hospitals (n = 99). The second is responsible for curative care, through Provincial and General Hospitals (n = 34), while the tertiary level, provides specialist care through Central and University Teaching Hospitals (n = 8) [23, 29]. Less than 4% of the population has private health insurance [32].

Over the past two decades, the Zambian Government has formulated strategies to address the most pressing healthcare challenges, such as access to basic care, infrastructure deficiencies, human resource shortages and the high dual burden of communicable and non-communicable diseases [20, 31]. Firstly, in 2001, Zambia was one of 54 African Union (AU) countries that adopted the Abuja declaration, pledging to work towards allocating at least 15% of total government expenditure to healthcare [33]. Secondly, in 2006, Zambia abolished healthcare user fees for rural patients, as the first step to free basic care for all [34]. Thirdly, from 2008, in line with the Ouagadougou Declaration, Zambia introduced mechanisms for community participation in healthcare provision, through the recruitment of Community Health Assistants (CHAs) and local volunteers [22]. Fourthly, since 2011, there has been commitment to addressing Zambia's human resources for health (HRH) shortfalls, through the National HRH Strategic Plan 2011-2015 and the 2013-2016 National Training Operational Plan (NTOP) [22, 29]. Also in 2011, the National Healthcare Standards (NHCS) were developed, to improve the quality of service delivery in health facilities. Medical imaging was identified as a specific service to be strengthened [35]. Fifthly, Zambia recently launched its seventh National Health Strategic Plan (NHSP), covering the period 2017-2021, and committing to UHC in the broad context of SDG realization [22, 29]. Also in 2017, the Zambia Colleges of Medicine and Surgery (ZACOMS) was established under Levy Mwanawasa Medical University (LMMU), in a bid to advance professional training of medical specialists and address the deficits in skilled Human Resources for Health (HRH) [36]. A ZACOMS Radiology-training program was launched in collaboration with the University of Rochester School of Medicine (URMC), Lusaka APEX Medical University and the Zambian Ministry of Health [37]. Sixthly, in 2018, the Social Health Insurance (SHI) Act No 2 of 2018 was passed, increasing health funding and promoting UHC [22, 38].

These initiatives have borne fruit, as reflected by the 69% decline in maternal mortality (729 vs. 224/100000 live births) between 2001 and 2015, as well as the 59% reduction in infant mortality (110 vs. 45/1000 live births) between 2000 and 2014 [19, 22, 23] Furthermore, HIV prevalence rates decreased 27% (15.7% vs. 11.5%) between 2000 and 2017 [20, 22], under-five mortality declined 32% (110 vs. 75/1000 live births) between 2005 and 2014 [19, 23] and hospital malaria fatalities decreased 22% (24.6 vs 19/1000 admissions) between 2014 and 2016 [22]. Infrastructure has been improved, with the University Teaching Hospital (UTH) and mammography units [22].

With a view to providing reference data for healthcare planning in low and middle-income countries (LMICs), a systematic evaluation of diagnostic radiology resources in African countries is being conducted by the Division of Radiodiagnosis of the Faculty of Medicine and Health Sciences at Stellenbosch University. Recently published data from South Africa, an upper middle-income country, revealed stark disparities between the public and private healthcare sectors, as well as geographical disparities within the public sector [9]. The analysis in Tanzania, a low-income African country, showed that public sector equipment resources were relatively equitably distributed across geographic regions, but the number of imaging units per million people is lower than WHO guidelines [8]. The Zimbabwean analysis revealed a strong urban private sector contribution to imaging resources [39]. There has been no comprehensive analysis of the radiology equipment and personnel resources in Zambia. Such a study will contribute to, and supplement, medium-term planning initiatives and provide a basis for assessing the status of UHC efforts in medical imaging. The primary aim was to conduct a comprehensive audit of licensed Zambian diagnostic imaging equipment and registered healthcare personnel. The secondary aim was to compare Zambian imaging resources with WHO guidelines on basic imaging equipment and recently published data from South Africa, Tanzania and Zimbabwe. The association between imaging resources, national economic indicators, and SDG health parameters for Zambia, South Africa, Tanzania and Zimbabwe was also assessed.

## Methods

This was a detailed audit of licensed Zambian imaging resources, conducted in Lusaka, Zambia, in October 2018, using information contained within the official registry databases of the Radiation Protection Authority (RPA) and the Health Professions Council of Zambia (HPCZ). The RPA, established in 2005 by the Government of the Republic of Zambia (GRZ), maintains a comprehensive inventory of the country's registered medical imaging equipment [40]. Data on general radiography (GR), fluoroscopy (FL), mammography (MM), computerized tomography (CT) and digital subtraction angiography (DSA) units were retrieved. Magnetic resonance imaging (MRI) and radioisotope equipment (single-photon emission computed tomography) data, which are not RPA registered, were obtained from the Nuclear Medicine and Radiology Departments of the University Teaching Hospital. All equipment data were captured on a customized Microsoft (MS) Excel spreadsheet stratified by imaging modality, provincial zone and healthcare sector. Ultrasound equipment, which is not licensed at RPA, was excluded.

The HPCZ, established in 2009 by the GRZ, maintains an annually updated register of the country's healthcare practitioners, including licensed radiation workers [35]. Data on imaging personnel, including Radiologists, Registrars, Nuclear Physicians, Radiographers, Radiography Technologists, Medical Physicists, Sonographers and Radiation Therapists were captured on a customized MS Excel spread sheet and stratified by the United Nations International Standard Classification of Occupations (ISCO), provincial placement and healthcare sector affiliation. For each imaging modality and category of healthcare worker, resources per million people were calculated for the country as a whole, by geographic region and by healthcare sector, based on the Central Statistics Office (CSO) of Zambia 2017 population data.

Equipment data were compared with the WHO guidelines on basic diagnostic imaging equipment requirements, and with recently published South African, Tanzanian and Zimbabwean country-level data [8, 9, 39]. For the calculation of equipment units per million people in the public and private sectors, respectively, it was assumed that 96% of the population (15.7 million people) were dependent on public sector facilities and 4% (0.66 million people) had direct access to private services [23, 32]. Aggregated data from recent World Bank and WHO resources were used for comparison of key healthcare, economic and SDG indicators [26, 27, 29, 41].

**Ethics approval:** the study was approved by the Health Research Ethics Committee (HREC) of the Faculty of Medicine and Health Sciences of Stellenbosch University, reference S18/05/104 and the University of Zambia Biomedical Research Ethics Committee (UNZABREC), reference 016-07-18. Authority to conduct the research was also obtained from the Zambian National Health Research Authority (NHRA) on 15 August 2018.

## Results

Table 1 and Table 2 respectively reflect Zambia's diagnostic imaging equipment and personnel resources. Comparisons of Zambian, Tanzanian, Zimbabwean and South African radiology equipment resources and health/economic indicators are shown in Table 3 and Table 4 respectively.

**Table 1:** Zambian diagnostic radiology equipment resources per million population by modality, province and by healthcare sector (Units/106 people)

Province (Population) (x 106)	Population density (/Km2)	GR	FL	MM	CT	MRI	DSA	SPECT
		Public [Private] {Total}	Public [Private} {Total}	Public [Private] {Total}	Public [Private] {Total}	Public [Private] {Total}	Public {Total}	Public {Total}
**Central** (1.59)	16.9	11(7.2) [4(67)] {15(9.4)}	0 [0] {0}	1 (0.66) [0] {1(0.63)}	0 [0] {0}	0 [0] {0}	0 {0}	0 {0}
**Copperbelt** (2.48)	79.2	19 (7.98) [14 (141)] {33 (13.3)}	1(.42) [0] {1(0.40)}	2 (0.84) [0] {2(0.81)}	2 (0.84) [1(10)] {3(1.21)}	0 [1 (10)] {1 (0.4)}	0 {0}	0 {0}
**Eastern** (0.91)	37.1	20 (10.9) [0 (0)] {20 (10.5)}	1 (0.55) [0] {1(0.52)}	1 (0.55) [0] {1(0.52)}	1(0.55) [0] {1(0.52)}	0 [0] {0}	0 {0}	0 {0}
**Luapula** (1.19)	23.4	8 (7.02) [0 (0)] {8 (6.7)}	0 [0] {0}	1 (0.88) [0] {1(0.84)}	0 [0] {0}	0 [0] {0}	0 {0}	0 {0}
**Lusaka** (3.01)	137.1	62 (21.5) [28 (233)] {90 (29.9)}	2 (0.69) [0] {2(0.66)}	6 (2.07) [4 (33.3)] {10(3.3)}	3(1.04) [3 (8.3)] {6(1.99)}	1(0.34) [2 (16.7)] {3(0.99)}	1 (0.34) {1(0.33)}	1(0.34) {1 (0.33)}
**Muchinga** (0.97)	11.1	7 (7.5) [0 (0)] {7 (7.21)}	0 [0] {0}	1 (1.07) [0] {1(1.03)}	0 [0] {0}	0 [0] {0}	0 {0}	0 {0}
**Northern** (1.39)	17.9	9 (6.77) [0 (0)]] {9 {(6.5)}	2(1.5) [0] {2(1.44)}	1 (0.75) [0] {1(0.72)}	1(0.75) [0] {1(0.72)}	0 [0] {0}	0 {0}	0 {0}
**North-Western** (0.88)	6.9	20 (23.8) [5 (125)] {25 (28.4)}	1(1.19) [0]] {1(1.14)}	1 (1.19) [0] {1(1.13)}	0 [1 (25)] {0}	0 [0] {0}	0 {0}	0 {0}
**Southern** (1.96)	23.1	14 (7.45) [3 (37.5)] {17 (8.7)}	0 [0] {0}	1(0.53) [0] {1(0.51)}	1 (0.53) [0] {1(0.51)}	0 [0] {0}	0 {0}	0 {0}
**Western** (1.02)	8.1	11 (11.1) [0 (0)] {11 (10.8)}	2(2.02) [0] {2(1.96)}	1 (1.02) [0] {1(0.98)}	0 [0] {0}	0 [0] {0}	0 {0}	0 {0}
**TOTAL (16.4)**	**21.8**	**181 (11.5) [54 (82)] {235(14.3)}**	**9 (0.57) [0] {9(0.55)}**	**16 (1.02) [4 (6.1)] {20(1.22)}**	**8 (0.51) [5 (7.65)] {13(0.79)}**	**1 (0.06) [3 (4.5)] {4(0.24)}**	**1 (0.064) {1(0.06)}**	**1 (0.064) {1(0.06)}**

GR: General Radiography, FL: Fluoroscopy, MM: Mammography, CT: Computed Tomography, MRI: Magnetic Resonance Imaging, DSA: Digital Subtraction Angiography, SPECT: Single Photon Emission computed tomography

**Table 2:** Zambian diagnostic radiology Human resources per million population by modality, province and by healthcare sector (Personnel/ 106 people)

Province (Population) (x 106)	RadiologistsISCO 2212	Registrars (STP)ISCO 2212	Nuclear PhysiciansISCO 2212	RadiographersISCO 3211	Radiography TechnologistsISCO 3211	Medical Physicists ISCO 2212	Sonographers ISCO 3211	Radiation TherapistsISCO 3211
	Public [Private] {Total}	Public [Private] {Total}	Public {Total}	Public [Private] {Total}	Public [Private] {Total}	Public {Total}	Public {Total}	Public {Total}
**Central** (1.59)	0 [0] {0}	0 [0] {0}	0 {0}	1 (0.65) [0] {1(0.63)}	43 (28.1) [2 (33.3)] {45(28.3)}	0 {0}	0 {0}	0 {0}
**Copperbelt** (2.48)	3 (1.26) [1 (10.1)] {4(1.61)}	0 [0] {0}	0 {0}	4 (1.68) [0] {4(1.61)}	87 (36.6) [33 (333)] {120(48.4)}	0 {0}	3(1.26) {3 (1.2)}	0 {0}
**Eastern** (0.91)	0 [0] {0}	0 [0] {0}	0 {0}	1 (0.55) [1(12.5)] {2(1.04)}	44 (24) [4 (50)] {48(25.1)}	0 {0}	0 {0}	0 {0}
**Luapula** (1.19)	0 [0] {0}	0 [0] {0}	0 {0}	2 (1.75) [0] {2(1.68)}	22 (19.3) [0] {22(18.4)}	0 {0	0 {0}	0 {0}
**Lusaka** (3.01)	4 (1.38) [1 (8.33)] {5(1.66)}	5 (1.73) [1(8.33)] {6(1.99)}	2(0.69) {2(0.7)}	13 (4.49) [5(41.)] {18(5.9)}	315 (108.9) [59(491.)] {374(124)}	3 (1.04) {3 (1.0)}	5 (1.66) {5 (1.66)}	12 (4.15) {12(3.99)}
**Muchinga** (0.97)	0 [0] {0}	0 [0] {0}	0 {0}	1 (1.07) [0] {1(1.03)}	25 (26.9) [1 (25)] {26(26.8)}	0 {0}	0 {0}	0 {0}
**Northern** (1.39)	0 [0] {0}	0 [0] {0}	0 {0}	0 [0 {0}	21 (15.7) [0] {21(15.1)}	0 {0}	0 {0}	0 {0}
**North-Western** (0.88)	0 [0] {0}	0 [0] {0}	0 {0}	3 (3.57) [0]] {3 (3.4)}	46 (57.4) [4 (100)] {50(56.8)}	0 {0}	1 (1.13) {1 (1.13)}	0 {0}
**Southern** (1.96)	1 (0.53) [0] {1 (0.51)}	0 [0] {0}	0 {0]	1 (0.53) [0] {1 (0.51)}	97 (51.6) [1(12.5)] {98 (50)}	0 {0}	0 0}	0 {0}
**Western** (1.02)	0 [0] {0}	0 [0] {0}	0 {0}	0 [0] {0}	34 (34.3) [0] {34 (33)}	0 {0}	1 (1.01) {1 (0.98)}	0 {0}
**TOTAL** (16.4)	**8 (0.51) [2 (3.03)] {10(0.61)}**	**5 (0.32) [1 (1.52)] {6 (0.37})**	**2(0.3) {2(0.12)}**	**26 (1.65) [6(9.09)] {32(1.9)}**	**734 (46.7) [104(158)] {838(51.)}**	**3 (0.19) {3 (0.18)}**	**8 (0.51) {8 (0.49)}**	**12 0.76) {12(0.7}**

ISCO: International Standard Classification of Occupations, STP: Speciality Training Program

**Table 3:** Comparison of registered radiological equipment resources in Zambia, Tanzania, Zimbabwe and South Africa by modality and health sector

Country	GR (Units / 106 People)			FL (Units / 106 People)			MM (Units / 106 People)			CT (Units / 106 People)			MRI (Units / 106 People)		
	Total [Public] (Private)	Lowest: Highest Regional Density in Public Sector	Public: Private	Total [Public] (Private)	Lowest: Highest Regional Density in Public Sector	Public: Private	Total [Public] (Private)	Lowest: Highest Regional Density in Public Sector	Public: Private	Total [Public] (Private)	Lowest: Highest Regional Density In Public Sector	Public: Private	Total Public (Private)	Lowest: Highest Regional Density in Public Sector	Public: Private
Tanzania	9 [6] (26)	1:2.2	1:5	1 [1] (1.9)	1:2	1:2	0.3 [0.2] (0.6)	0:0.5	1:3	0.42 [0.08] (2.15)	0:0.2	1:27	0.09 [0.05] (0.27)	0:0.24	1:5
Zimbabwe	26 [11] (16)	1:5	1:16	0.8 [0.1] (6.9)	0: 0.5	1:69	0.8 [0.2] (6.1)	0:1.7	1:31	1.5 [0.6] (9)	0:3.4	1:16	0.5 [0.2] (3.1)	0: 1.7	1:15
**Zambia**	14.3 [11.5] (82)	1: 3.5	7.1:1	0.55 [0.57] (0)	0: 2	2: 0	1.22 [1.02] (6.1)	1:5.58	2.75: 1	0.79 [0.5]1 (7.65)	0: 3	1.6: 1	0.24 [0.06] (4.5)	0: 1	1:3
South Africa	35 [20] (104)	1:2.5	1:5	6.6 [2.5] (26.8)	1:9	1:11	5 [1.3] (22.3)	0:2.6	1:17	5 [1.7] (20.7)	1:6.8	1:12	2.9 [0.3] (15.1)	0:0.8	1:46

GR: General Radiography, FL: Fluoroscopy, MM: Mammography, CT: Computed Tomography, MRI: Magnetic Resonance Imaging

**Table 4:** Comparison of Economic, Demographic and Healthcare Indicator Data: Zambia vs Tanzania vs Zimbabwe vs South Africa

(a) Economics:	Tanzania	Zimbabwe	Zambia	South Africa
World Bank Income Country Classification	Low Income	Low income	Lower middle income	Upper middle income
GNI per capita, calculated using the World Bank Atlas method	Less than $1,025	Less than $995	Between $1,026 - $3,995	Between $3,996 - $12,375
Actual GNI per capita, calculated using the World Bank Atlas method * (2017)	$910	$910	$1 300	$5 430
GDP in billion USD (annual growth) (2017)	52.09 (7.1%)	17.85 (3.4%)	25.81 (4.1%)	349.42 (1.3%)
Health expenditures as a percentage (%) of GDP of GDP (2016)	4,10%	9,40%	5%	8,10%
Health expenditure per capita in USD (2016)	32,09	93,94	56,54	428,18
Health Insurance coverage (% of the total population insured)	**16,6**	**10**	**4**	**17**
**(b) Demographics:**	**Tanzania**	**Zimbabwe**	**Zambia**	**South Africa**
Population, Million People (Annual growth rate) (2017)	57.31 (3.1%)	16.53 (2.3%)	16.4 (3.0%)	56.72 (1.2%)
Area [x103 Km2]] (Population Density)	890.1 (64.7)	390.8 (42.7)	752.6 (23)	1219.1 (46.8)
Urban population as a percentage (% )of total population(2018)	33,80%	32,20%	43.5%	66,40%
**(c) Selected Heath Indicators:**	**Tanzania**	**Zimbabwe**	**Zambia**	**South Africa**
Life expectancy at birth, total (years)	66	61	62	63
Maternal mortality ratio (national estimate, per 100,000 live births) (2015 est.)	398	443	224	138
Births attended by skilled health staff (% of total)	64%	78%	63%	97%
Skilled health professional density per 10,000 population (2005-2015)	4,6	12,7	9,7	58,8
Mortality rate, under-5 (per 1,000 live births)	54	50	96	60
Death rate (per 1,000 population [2018 est.])	7,5	9,9	12	9,3
Immunization, measles (% of children ages 12-23 months)	99	90	96	60
Prevalence of HIV, total (% of population ages 15-49)	4,5	13,3	11,5	18,8
Tuberculosis incidence (per 100 000 population) (2016)	287	208		

**Overview:** there are 283 registered equipment units and 913 registered workers in the diagnostic imaging domain, nationally. Just over half of all equipment (153/283 units, 54 %) and almost two thirds of all radiation workers (556/913, 61%) are in two provinces (Lusaka and Copperbelt), serving one third of the population (5.49/16.4, 33.5%). Approximately three-quarters of the national equipment inventory (212/283 units, 75%) and nearly ninety percent of registered radiation workers (800/913, 88%) are in the public sector, serving 96% of the population. There is a cost-driven hierarchy of access to public sector imaging equipment, with more affordable units, such as GR, being more accessible. Accordingly, GR is the most widely available modality across the ten administrative provinces, with installations from District Hospital level. Public sector MM units are present in all ten provinces. CT is available at some Provincial/Central Hospitals and MRI at a single Teaching Hospital. National diagnostic imaging capacity in the private sector is generally higher than that in the public sector. The overall private sector resource contribution is 23% of national equipment units (66/283 units) and 12% (113/913 people) of human resources, but there is striking inhomogeneity in geographic distribution of private-sector resources.

**Public-sector equipment:** the best-resourced provinces are the most densely populated, being Lusaka and Copperbelt. Lusaka is the only province with the full spectrum of imaging modalities, while Copperbelt lacks MRI, digital subtraction angiography (DSA) and nuclear imaging.

***General radiography (GR):*** GR is available in all provinces and the units represent 85% of public sector imaging equipment. Overall availability (14.3 units/10^6^ people), is below the WHO guideline of 20 units/10^6^ people. Only two provinces, Lusaka (29.9 units/10^6^) and North-Western (28.4 units/10^6^) meet the WHO benchmark. Of note, North-Western has the lowest population density (6.9 million people/km^2^) and the largest surface area (125.3 km^2^). The best resourced province (Lusaka) has 3.5 times the units of the least resourced (Northern).

***Fluoroscopy (FL):*** FL is available in six provinces (Copperbelt, Eastern, Lusaka, Northern, North-Western and Western), with Western Province (2.02/10^6^) the best resourced. FL units represent 4% of public-sector imaging equipment. More than one-third of the population (5.7 x 10^6^ people, 35%) has no direct access to public sector FL.

***Mammography (MM):*** mammography is available in all ten provinces with Lusaka (3.3 units/10^6^ people) the best resourced. Mammography units represent 5% of public-sector imaging equipment.

***Computed tomography (CT):*** CT units represent 3.7% of public-sector imaging equipment. Two-thirds of the population (10.7 x 10^6^ people, 66%) have direct access to the modality. The ratio of GR to CT units is 18:1, nationally.

***Magnetic resonance imaging (MRI):*** the single public sector MRI unit, in Lusaka, represents 0.5% of the national imaging equipment. The ratio of public sector CT to MRI units is 8:1.

***Other modalities:*** DSA and single-photon emission computed tomography (SPECT) are only available in Lusaka. There are no positron emission tomography (PET) units in the country.

**Private-sector equipment:** almost a quarter (66/278; 23.7%) of the national imaging equipment inventory is in the private sector, and thus freely available to approximately 4% of the population.

***GR:*** GR units represent 68% (45/66) of all private sector equipment. Overall access to the modality (82 units/10^6^ people) is 4-times the WHO recommendation, with a 7-fold disparity between the private and public sectors (11.5 vs 82 units/10^6^ people). While almost one quarter of GR units nationally (54/235, 23%) are in the private sector, these are distributed across just 5 provinces, with more than eighty percent of resources (42/54 units, 83%) in two provinces (Lusaka and Copperbelt).

***MM:*** private MM is only available in Lusaka and the total units represent 6% of private imaging equipment resources, nationally (4/66). There is an overall 6-fold disparity between public and private sector access, with private resources representing less than a quarter (4/20, 20%) of the national mammography inventory.

***CT:*** private CT units are available in 3 provinces (Lusaka, Copperbelt and North-Western) and represent 7.5% of private imaging resources, nationally (5/66). There is an overall 15-fold disparity between private- and public-sector access, with private resources representing almost forty percent (5/13, 38%) of CT units nationally. The ratio of plain radiography to CT units in the private sector is 11:1.

***MRI:*** available in two provinces (Lusaka and Copperbelt), private units represent 4.5% (3/66) of private imaging resources nationally. There is a 75-fold disparity between private and public-sector access, with private resources representing three-quarters (3/4, 75%) of MRI units, nationally. There exists a 1.6:1 ratio between private-sector CT and MR units.

**Public sector personnel:** diploma-trained (3 years) Radiography Technologists (RTs) (n = 734) are the most abundant human resource, constituting 93% (734/788) of the public-sector imaging workforce, with representation across all provinces and overall availability of 47/10^6^ people. Eighty-seven percent (734/838) of the national cohort are employed in the public sector, while more than forty percent (315/734, 43%) of public-sector RTs are in Lusaka, contributing to a 7-fold differential between the best- (Lusaka, 109/10^6^ people) and least-resourced province (Northern, 15.7/106 people). Degree-trained (4 years) Radiographers (n = 26) are the second most abundant human resource, but constitute just 3% (26/788) of the public-sector workforce, with representation in 8 provinces and overall availability of 1.7/10^6^ people. Half the public-sector cohort (13/26, 50%) is employed in Lusaka. The RT: Radiographer ratio is 28:1, while that of radiation worker (Radiographer and RT) to public-sector equipment unit is 3.6:1, nationally. Radiologists (n = 8; 0.5/10^6^ people) constitute 1% of the public-sector workforce and are largely in two provinces (Lusaka, Copperbelt), with a single practitioner in the Southern Province. Eighty percent of the country's Radiologists are in this sector, which has a 95:1 Radiographic staff: Radiologist differential. All Medical Physicists (n = 3; 0.18/10^6^ people) and Nuclear Physicians (n = 2; 0.12/10^6^ people) are in the public-sector, and based in Lusaka.

**Private sector personnel:** more than ten percent (113/913; 12.3%) of national personnel resources are in the private sector and thus freely available to approximately 4% of the population. The private: public disparity is greatest in the categories of Radiologist and Radiographer, where approximately one-fifth of national resources are in this sector.

**Comparison of Zambian, Tanzanian, Zimbabwean and SA radiology equipment resources and health/economic indicators:** public sector imaging equipment resources broadly reflect national per capita healthcare expenditure (Tanzania: $32.09, Zimbabwe: $93.84, Zambia: $56.56 and South Africa: $428.18 [41], such that the higher the national expenditure, the greater the resources. Of note, relative GR equipment resources within the public sector are closely aligned with World Bank income grouping (Table 3 and Table 4). Zimbabwe and Zambia have a less equitable distribution of GR equipment than Tanzania and South Africa. Despite having the lowest per capita healthcare expenditure and public sector resources, Tanzania has the most equitable distribution of basic equipment, and the lowest discrepancy in access between the public and private sectors [8]. Although Zambian and Zimbabwean imaging resources exceed those of Tanzania, the latter generally has superior healthcare indicators. For instance, Tanzanians have higher life expectancy at birth (66 years) than South Africa (63 years), Zambia (62 years) and Zimbabwe (61 years). South Africa and Zimbabwe have formidable national private sector resource contributions compared to Tanzania and Zambia. Of note, health insurance coverage is low in Zambia (4%), compared to Zimbabwe (10%) ,Tanzania (16.6%) and South Africa (17%) [8, 9, 32, 39].

## Discussion

Our study represents the first comprehensive review of Zambian diagnostic imaging capacity. To the best of our knowledge, it also represents the first unifying assessment of registered equipment and human resources for imaging in a low middle-income country. It thus makes an important contribution to the current discourse on global imaging resources. Such analyses provide useful baseline data for national healthcare planning. Of note, during the course of this project, the Zambian government adopted the Social Health Insurance (SHI) Act, aimed at increasing national healthcare funding and promoting UHC [22, 29]. This context underscores the importance of this analysis, as it contributes to the dialogue on radiological resources required for effective UHC.

Zambia's public-sector radiological equipment and human resources could be more equitably distributed across administrative provinces. Currently, the densely populated, predominantly urban provinces of Lusaka and the Copperbelt are substantially better resourced than sparsely populated regions such as Muchinga, Northern and Southern Provinces. There is thus an opportunity for increased, coordinated, central government control of services. The 11.5 GR units/10^6^ people in the public sector is lower than the 20 units/10^6^ people recommended by the WHO. Just two provinces, Lusaka (21.5/10^6^) and North-Western (23.8/10^6^) achieved this threshold. The overall shortfall of approximately 8 GR units/10^6^ people equates to a total deficit of approximately 100 units nationally, informing forward planning. This study also defines the optimum location of any new GR units. A coordinated drive to attain the WHO benchmark in the remaining eight provinces would contribute substantially to the achievement of equitable imaging access.

In resource-limited environments, notwithstanding the availability of diagnostic imaging equipment, the quality and safety of procedures may be compromised by a paucity of qualified imaging personnel [11, 22, 29]. This is especially true in sparsely populated rural areas [1, 8, 15]. Our finding that more than 40% of public-sector radiographic staff and all Medical Physicists are in Lusaka highlights the challenge of achieving a more equitable distribution of Zambian imaging personnel. By defining the national shortfall and the preferred distribution of basic radiography units going forward, the accompanying requirement for qualified radiographic staff can be extrapolated. Of note, enhanced access to basic imaging services increases the need for accurate image interpretation. It is thus likely that radiographic staff in rural areas will require appropriate training to assume an extended role, inclusive of basic imaging interpretation. It is therefore commendable that the Zambian MoH is actively pursuing mechanisms for the certification or training of these professionals in order to help to address the deficits [22, 36, 37]. Such local training ingenuities should be supported, as should the initiatives that enable Zambians to train abroad and return with these much sought-after skillsets. Our finding that 23% of equipment units are in the private sector, but only 4% of the population has medical insurance suggests that the private sector is partially fuelled by “out-of-pocket ” expenditure by those who have no cover.

The strength of this quantitative work is its foundation on the official RPA database of registered diagnostic imaging equipment, and the official HPCZ database of registered healthcare workers. A limitation is the failure to include a qualitative component of equipment functionality. This may have introduced inherent positive bias in the assessment of equipment resources, particularly in the public sector. An additional limitation is the exclusion of diagnostic ultrasound equipment, since it does not involve ionizing radiation and is not RPA-registered. This constraint is common to all contemporary studies of national diagnostic imaging resources and is a major drawback in the appraisal of the imaging capacity in LMICs, where ultrasound has the ability to perform a fundamental role. Going forward, chronicling of all diagnostic ultrasound equipment will facilitate healthcare planning.

## Conclusion

To achieve equitable imaging access, pivotal for UHC, Zambia will need a more homogeneous distribution of specialised radiological resources tailored to remedy disparities between healthcare sectors and geographical regions. Analyses of licenced radiology resources at country level can serve as a benchmark for medium-term radiological planning.

### What is known about this topic

- Estimates indicate that two-thirds of the world’s population lack adequate access to basic medical imaging services integral to universal health coverage;
- Limited country-level radiological resource statistics exist and there is scant appreciation of how such data reflect healthcare access, especially in lower middle-income African countries.

### What this study adds

- This study provides a comprehensive analysis of national diagnostic radiology equipment resources in a lower middle-income African country;
- Analyses of licenced radiology resources at country level help define national deficits and can serve as a benchmark for medium-term radiological planning.

## Competing interests

The authors declare no competing interests.
